# Supplementary material for: Comparative genomics provides new insights into the diversity, physiology, and sexuality of the only industrially exploited tremellomycete: Phaffia rhodozyma
Source: BMC Genomics. 2016 Nov 9;17:901. doi: 10.1186/s12864-016-3244-7 (PMC5103461; doi:10.1186/s12864-016-3244-7)
Supplement: Additional file 6: — List of orphan genes with links to PFAM (related to Additional file 1: Table S1). (ZIP 1428 kb) [file 12864_2016_3244_MOESM6_ESM.zip › PFAM_UNIQUE/G05227_P.html]

#                                                                            --- full sequence --- -------------- this domain -------------   hmm coord   ali coord   env coord
  
# target name        accession   tlen query name           accession   qlen   E-value  score  bias   #  of  c-Evalue  i-Evalue  score  bias  from    to  from    to  from    to  acc description of target
  
#------------------- ---------- ----- -------------------- ---------- ----- --------- ------ ----- --- --- --------- --------- ------ ----- ----- ----- ----- ----- ----- ----- ---- ---------------------
  
G05227\_P             -            173 DUF3984              PF13136.1    325       4.6    5.0   8.8   1   1    0.0099       5.9    4.7   8.8    43   103    37   106    13   141 0.54 - Protein of unknown function (DUF3984)  
G05227\_P             -            173 MAGE\_N               PF12440.3     96       5.1    6.1  11.2   1   2     0.001      0.75    8.8   6.9    36    89    26    80    16    86 0.88 - Melanoma associated antigen family N terminal  
G05227\_P             -            173 MAGE\_N               PF12440.3     96       5.1    6.1  11.2   2   2         2   1.5e+03   -1.8   0.1    62    62   117   117    89   142 0.55 - Melanoma associated antigen family N terminal
